# Commentary: The Efficacy of Nerve Growth Factor Antibody for the Treatment of Osteoarthritis Pain and Chronic Low-Back Pain: A Meta-analysis

**DOI:** 10.3389/fphar.2021.619344

**Published:** 2021-04-20

**Authors:** Rodrigo R. N. Rizzo, Michael A. Wewege, Hayley B. Leake, James H. McAuley

**Affiliations:** ^1^ Centre for Pain IMPACT, Neuroscience Research Australia, Sydney, NSW, Australia; ^2^ School of Health Sciences, University of NSW, Sydney, NSW, Australia; ^3^ IIMPACT in Health, University of SA, Adelaide, NSW, Australia

**Keywords:** anti-nerve growth factor antibody, osteoarthritis pain, chronic low-back pain, meta-analysis, systematic review

The recent article by Yang et al. (2020) is timely. Nerve growth factor (NGF) inhibitors may have benefits for chronic pain, and the U. S. Food and Drug Administration has recently accepted regulatory submission of tanezumab (an anti-NGF agent) for osteoarthritis. However, we have two major concerns with the review that question the validity of their results: 1) missing studies and 2) incorrect data extraction/analysis.

First, Yang et al. (2020) have missed several osteoarthritis trials that were eligible for inclusion in their review, for example, Schnitzer et al. (2015), Brown et al. (2013), and Brown et al. (2012). These trials are indexed in both Embase and PubMed (two of the databases searched by Yang et al. (2020)) and meet all inclusion criteria for the review. Brown et al. (2013) and Brown et al. (2012) are also found in the reference list of one of the included trials (Kivitz et al., 2013) indicating that Yang et al. (2020) failed to include these studies since they stated in the review that “*The references of the articles included were also searched in case of any additional studies not previously identified in the initial literature search.*”

In regard to trials for chronic low back pain, we have concerns about the decision by Yang et al. (2020) to restrict inclusion criteria to only studies that assessed pain intensity using the Western Ontario and McMaster Universities Arthritis Index (WOMAC). Justification for this decision is unclear; the WOMAC was designed to assess individuals with osteoarthritis (Bellamy, 2002) and it is rarely used in low back pain clinical trials (Chiarotto et al., 2018). By excluding studies that assessed pain intensity using measurement tools that are more common in back pain trials (e.g. visual analogue scale, numerical rating scale), several relevant trials have likely been missed. Probably, this was also the reason that Yang et al. (2020) only identified one study for low back pain. Interestingly, the one low back pain trial (Kivitz et al., 2013) included in the Yang et al. (2020) review did not actually use the WOMAC to measure pain intensity, despite the authors stating that it did. Kivitz et al. (2013), used the 11-point numerical rating scale, leading us to believe other eligible trials with similar outcomes should be included (Katz et al., 2011; Sanga et al., 2016).

**FIGURE 1:**
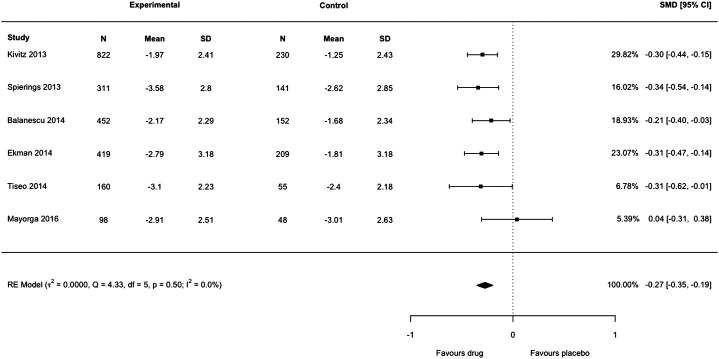
Forest plot of changes from baseline to checkpoint for pain. This is a re-analysis in which we transformed the standard error reported in individual studies to standard deviation. SMD = Standardised Mean Difference. CI = confidence Interval. N = number of participants.

Our second concern is that Yang et al. (2020) have incorrectly used the standard error (SE) in place of standard deviation (SD) as the arm-level measure of variance for each included trial. In Figure 4 of their article, anti-NGF interventions produced a substantial decrease in pain intensity compared to placebo: standardized mean difference −2.22 (95% confidence interval −3.44 to −0.99). Substantial heterogeneity and inconsistency were reported (*tau* ^2^ = 2.3248, *I* ^2^ *=* 99%). We noted that only Tiseo et al. (2014) had reported SD; all other studies reported SE but had not been converted to SD by Yang et al. (2020) before analysis. We re-extracted the data of each trial in Figure 4 of the authors’ review, transformed SE to SD for each arm in five studies, then lumped the intervention arms together using recommendations from the *Cochrane Handbook*, emulating the process undertaken by Yang et al. (2020). We then reproduced the meta-analysis with the correct data using the *metafor* package in R which resulted in a markedly smaller effect size: standardized mean difference −0.27 (95% confidence interval −0.35 to −0.19) (Figure 1 of this letter). We detected no heterogeneity or inconsistency (*tau* ^2^ = 0.0, *I* ^2^ *=* 0%). There is a substantial discrepancy between the results reported by Yang et al. (2020) and our re-analysis. The American College of Physicians Clinical Practice Guideline by Chou et al. (2017) suggests a large/substantial reduction in pain intensity corresponds to a standardized mean difference >0.8 (approximately >2 points on a 0 to 10-point scale). The results from Yang et al. (2020) show evidence for an effect magnitude (−2.22) that is extremely large based on recommended thresholds for clinically important differences (Chou et al., 2017), which infers that anti-NGF drugs are a game-changing treatment for reducing pain. Our re-analysis with the appropriate data shows the correct result is −0.27, eight times smaller than the original effect and one that is unlikely to be considered slight/small (<0.5), and therefore meaningful, by Chou et al. (2017). We highlight the importance of checking data extraction carefully and scrutinizing the results of meta-analyses. Data and code of our reanalysis are available at https://osf.io/tp4e6/.

In conclusion, we urge readers to be cautious of the quality and validity of Yang et al. (2020)’s review. The review misses several important trials and contains extraction/synthesis errors in the meta-analysis which resulted in authors overstating the efficacy of anti-NGF for patients with osteoarthritis and chronic low back pain.
