# Supplementary material for: Commentary: The Efficacy of Nerve Growth Factor Antibody for the Treatment of Osteoarthritis Pain and Chronic Low-Back Pain: A Meta-analysis
Source: Front Pharmacol. 2021 Apr 20;12:619344. doi: 10.3389/fphar.2021.619344 (PMC8108005; doi:10.3389/fphar.2021.619344)
Supplement: Supplementary file 2 [file datasheet1.docx]

**Commentary: Yang et al. 2020 'The efficacy of nerve growth factor antibody for the treatment of osteoarthritis pain and chronic low-back pain: A meta-analysis', Frontiers in Pharmacology**

- Excel file and R codes can be accessed with this link: <https://figshare.com/s/270e03615f677566ae64>
- We also provide the codes in this Word doc:

## Yang 2020 - Analysis ####

## Set-up ####

library(readxl)

library(dplyr)

library(metafor)

setwd() # Adjust as required

data <- read_xlsx("Analysis.xlsx",

sheet = "Yang 2020",

range = "A1:H7")

str(data)

View(data)

## Recreate Figure 4 - WOMAC Pain subscale ####

ma_pain <- rma(data = data,

m1i = Exp_mean,

sd1i = Exp_SD,

n1i = Exp_N,

m2i = Cont_mean,

sd2i = Cont_SD,

n2i = Cont_N,

weighted = TRUE,

measure = "SMD",

method = "DL",

slab = Study)

ma_pain

forest(ma_pain,

addfit = TRUE,

showweights = TRUE,

xlim = c(-18, 8),

at = c(-6, -4, -2, 0, 2, 4, 6),

ilab = cbind(data$Exp_N,

data$Exp_mean,

data$Exp_SD,

data$Cont_N,

data$Cont_mean,

data$Cont_SD),

ilab.xpos = c(-13.5, -12, -10.5, -9, -7.5, -6),

rows = (6:1),

cex = 0.75,

mlab = "",

psize = 1,

xlab = "Favours drug Favours placebo")

op <- par(cex = 0.75, font = 2)

text(c(-13.5, -12, -10.5, -9, -7.5, -6), 6.5, c("N", "Mean", "SD", "N", "Mean", "SD"))

text(c(-12, -7.5), 7.5, c("Experimental", "Control"))

text(-18, 6.5, "Study", pos=4)

text(0, 7.5, "SMD [95% CI]", pos=2)

text(-18, -1, pos = 4, cex = 1,

bquote(paste("RE Model (", tau^2, " = ",

.(formatC(ma_pain$tau2, digits=4, format="f")), ", Q = ",

.(formatC(ma_pain$QE, digits=2, format="f")), ", df = ",

.(ma_pain$k - ma_pain$p), ", p = ",

.(formatC(ma_pain$QEp, digits=2, format="f")), "; ", I^2, " = ",

.(formatC(ma_pain$I2, digits=1, format="f")), "%)")))

Figure4_Yang <- recordPlot()

## Correct Figure 4 - Pain intensity (SMD) ####

data <- read_xlsx("Analysis.xlsx",

sheet = "Yang 2020",

range = "A10:O31")

data <- data %>% filter(Recalculated == 1)

View(data)

ma_pain <- rma(data = data,

m1i = Exp_mean,

sd1i = Exp_SD,

n1i = Exp_N,

m2i = Cont_mean,

sd2i = Cont_SD,

n2i = Cont_N,

weighted = TRUE,

measure = "SMD",

method = "DL",

slab = Study)

ma_pain

dev.off()

forest(ma_pain,

addfit = TRUE,

showweights = TRUE,

xlim = c(-5, 2),

at = c(-1, 0, 1),

ilab = cbind(data$Exp_N,

data$Exp_mean,

data$Exp_SD,

data$Cont_N,

data$Cont_mean,

data$Cont_SD),

ilab.xpos = c(-4, -3.5, -3, -2.25, -1.75, -1.25),

rows = (6:1),

cex = 0.8,

mlab = "",

psize = 1,

xlab = "Favours drug Favours placebo")

op <- par(cex = 0.8, font = 2)

text(c(-4, -3.5, -3, -2.25, -1.75, -1.25), 6.5, c("N", "Mean", "SD", "N", "Mean", "SD"))

text(c(-3.5, -1.75), 7.5, c("Experimental", "Control"))

text(-5, 6.5, "Study", pos=4)

text(2, 7.5, "SMD [95% CI]", pos=2)

text(-5, -1, pos = 4, cex = 1,

bquote(paste("RE Model (", tau^2, " = ",

.(formatC(ma_pain$tau2, digits=4, format="f")), ", Q = ",

.(formatC(ma_pain$QE, digits=2, format="f")), ", df = ",

.(ma_pain$k - ma_pain$p), ", p = ",

.(formatC(ma_pain$QEp, digits=2, format="f")), "; ", I^2, " = ",

.(formatC(ma_pain$I2, digits=1, format="f")), "%)")))

Figure4_Recreate_SMD <- recordPlot()

## Correct Figure 4 - Pain intensity (MD) ####

data <- read_xlsx("Analysis.xlsx",

sheet = "Yang 2020",

range = "A10:O31")

data <- data %>% filter(Recalculated == 1)

View(data)

ma_pain <- rma(data = data,

m1i = Exp_mean,

sd1i = Exp_SD,

n1i = Exp_N,

m2i = Cont_mean,

sd2i = Cont_SD,

n2i = Cont_N,

weighted = TRUE,

measure = "MD",

method = "DL",

slab = Study)

ma_pain

dev.off()

forest(ma_pain,

addfit = TRUE,

showweights = TRUE,

xlim = c(-18, 8),

at = c(-2, 0, 2),

ilab = cbind(data$Exp_N,

data$Exp_mean,

data$Exp_SD,

data$Cont_N,

data$Cont_mean,

data$Cont_SD),

ilab.xpos = c(-13.5, -12, -10.5, -9, -7.5, -6),

rows = (6:1),

cex = 0.75,

mlab = "",

psize = 1,

xlab = "Favours drug Favours placebo")

op <- par(cex = 0.75, font = 2)

text(c(-13.5, -12, -10.5, -9, -7.5, -6), 6.5, c("N", "Mean", "SD", "N", "Mean", "SD"))

text(c(-12, -7.5), 7.5, c("Experimental", "Control"))

text(-18, 6.5, "Study", pos=4)

text(0, 7.5, "MD [95% CI]", pos=2)

text(-18, -1, pos = 4, cex = 1,

bquote(paste("RE Model (", tau^2, " = ",

.(formatC(ma_pain$tau2, digits=4, format="f")), ", Q = ",

.(formatC(ma_pain$QE, digits=2, format="f")), ", df = ",

.(ma_pain$k - ma_pain$p), ", p = ",

.(formatC(ma_pain$QEp, digits=2, format="f")), "; ", I^2, " = ",

.(formatC(ma_pain$I2, digits=1, format="f")), "%)")))

Figure4_Recreate_MD <- recordPlot()
